# History of Asbestos Ban in Hong Kong

**DOI:** 10.3390/ijerph14111327

**Published:** 2017-10-31

**Authors:** Chun-Kwan Wong, Sabrina Hei-Man Wan, Ignatius Tak-Sun Yu

**Affiliations:** 1 Hong Kong Workers’ Health Centre, Room 1429 Beverley Commercial Centre, Tsimshatsui, Hong Kong, China; clive@hkwhc.org.hk (C.-K.W.); sabrina@hkwhc.org.hk (S.H.-M.W.); 2 Hong Kong Occupational & Environmental Health Academy, Room 1418 Beverley Commercial Centre, Tsimshatsui, Hong Kong, China

**Keywords:** asbestos ban, policy, compensation system

## Abstract

As millions of immigrants moved to Hong Kong (HK) from China in the recent decades, large amount of residential housings were built in the early years and a substantial proportion of those buildings used asbestos-containing materials (ACMs). Since the number of new cases of ARDs diagnosed has increased year by year since 1990’s, the remarkable increase of incidences had drawn the attention of the public and most importantly the HK government. It became one of the trigger points leading to asbestos ban in HK history. Comparatively, non-governmental organizations (NGOs), labor unions and patients’ self-help organizations demonstrated a more aggressive and proactive attitude than the HK government and have played a key role in the development of asbestos banning policy in HK. After numerous petitions and meetings with the government representatives by those parties in the past decade, the HK government eventually changed its attitude and started to consider terminating the endless threat from asbestos by amending the policy, and the new clause of legislation for banning of all forms of asbestos was enacted on 4 April 2014. Other than the restriction of asbestos use, the compensation system about ARDs has also made some great moves by the effort of those parties as well. Based on the experience we learnt through the years, efforts from different stakeholders including patients’ self-help organizations, NGOs, legislative councilors, and media power are absolutely essential to the success of progression and development in today’s asbestos banning in HK.

## 1. Introduction

Hong Kong (HK), a city located on the Southeast coast of China, is the first Special Administrative Region (SAR) established under the policy of “One Country, Two Systems” [1]. Before the sovereignty was handed back to China on 1 July 1997, HK had been one of the colonies of the United Kingdom since 1842 [2]. With plenty of resources and support provided by The British government, HK has become one of the most civilized cities and largest financial centers in the world [3].

Gifted with the wonderful geographical location in Asia-Pacific region and the renowned deep-water harbor between Hong Kong Island and Kowloon Peninsula, HK is also one of the biggest import and re-export centers on the globe, acting as a bridge to associate between Mainland China and other countries in Asia, Europe and Latin America [2,4]. HK’s GDP per capita was USD$43,500 in 2016, according to the HK government’s data, with the amount of USD$513.9 billion from import and USD$454.5 billion from re-export [5].

After a series of wars, natural disasters and socio-political movements led to millions of immigrants moving to HK from Mainland China. As a result, the population has increased dramatically in the recent decades. The population of HK jumped from 456,739 in 1911 to 3,129,648 in 1961 and 7,071,576 in 2011, over 15-fold in 100 years [6]. Along with the population explosion, large amount of public and private residential housings were built in 1940’s and 1950’s to satisfy the need. Similar to other developed countries in the world, a substantial proportion of those buildings built in the early years used asbestos-containing materials (ACMs); and the peak period of adopting large amount of asbestos for building construction was in 1960’s [7]. According to a previous study, the yearly use of asbestos per capita was about 8.84 kg/capita/year in 1960–1963, and this number dropped to less than 1 kg/capita/year after 1986 [7].

As there is neither asbestos ores nor asbestos or ACMs produced in HK, all the ACMs used in HK were imported. These ACMs in HK were commonly found as corrugated cement sheets for roofing, wall board, cement roof tiles, vinyl floor coverings and the stuffing inside cable trays [8]. Based on the statistics released by the Environmental Bureau of HK government in 2012, about 15,000 buildings in HK aged over 20 years, i.e., built before 1992, were believed to have asbestos-containing construction materials [9].

## 2. Materials and Methods

This paper is aimed to illustrate the process of total asbestos ban policy development in Hong Kong and the experience learned in the past decades.

Authors reviewed relevant documents and sources, including data released by local government, results from previous researches, position papers on asbestos issue submitted by different interested parties, and examined the factors leading to the asbestos ban and establishment of the compensation system for asbestos-related diseases.

## 3. Results and Discussion

### 3.1. Health Burden of Asbestos-Related Diseases and Legislation Systems about Asbestos in HK

Since the latency period of asbestos-related diseases (ARDs) can be as long as 40 years or longer, the number of new cases of ARDs diagnosed has increased year by year since 1990’s [7,10]. According to the data of Hong Kong Cancer Registry gathered by previous study, the age-standardized incidence rate of mesothelioma was less than 0.5 per million populations in 1976–1990. However, the rate has sharply increased in the mid-90s and reached to 2.5 in 2006 [7]. There were 36 and 96 confirmed new cases of asbestosis and mesothelioma respectively within 2006–2015, and 87% of them had employment record in construction industry [11]. According to the prediction by Chang et al. [10], the incidence of mesothelioma may attain a peak between the years 2010 to 2020. The remarkable increase of incidences had drawn the attention of the public, including non-governmental organizations (NGOs), industrial unions and patients’ self-help organizations, and most importantly the HK government. The growing social attention has had a great impact on the “Positive Non-interventionism” policy (a term used to define a policy and governance strategy, which means a government will generally not intervene business decisions so as to establish a “free-market”) which runs by the HK government since 1970’s [12], and became one of the trigger points leading to asbestos ban in HK history.

Because of the health burden to humans, activities on banning of asbestos were started by other countries in the world nearly 50 years ago and was firstly introduced by Denmark in 1972 [13]. As some scientists claimed that materials under the amphibole group including amosite (brown asbestos) and crocidolite (blue asbestos) are much more hazardous than that under serpentine group, i.e., chrysotile (white asbestos), the use of the former groups of asbestos were banned since 1996 by Environmental Protection Department (EPD) in Hong Kong [14,15]. Under Section 80 of Air Pollution Control Ordinance (Chapter 311 (cap.311) of the Hong Kong Legislation), a person shall not import into Hong Kong or sell any quantity of asbestos known as amosite or crocidolite or any substance or item made with or containing both [16]. In fact, the asbestos banning policy of HK was established and applied in several stages along the past decades. The other kind of asbestos such as chrysotile could still be imported, manufactured and exported after 1996 [16], and the health of HK residents was still threatened by the hazard of asbestos.

Cap.311 was not the first law to mention asbestos in HK history, but the first law to restrict the use of asbestos. Before that, the Factories and Industrial Undertakings (Asbestos) Special Regulations (Cap.59X) was initially introduced to the HK legal system in 1986 to oversee the work with asbestos carried out in industrial undertakings, but it was repealed and replaced by the new Factories and Industrial Undertakings (Asbestos) Regulation (Cap.59AD) since 1997 [17,18]. However, the introduction of Cap.311 set a precedent to the policy makers and the public. Based on similar principle, the enforcement of Hazardous Chemicals Control Ordinance (Cap.595), which regulates the manufacturing, export, import and use of chemicals especially those included in the regulation of the Rotterdam Convention on the Prior Informed Consent Procedure for Certain Hazardous Chemicals and Pesticides in International Trade, or the Stockholm Convention on Persistent Organic Pollutants, was introduced in 2008 [19]. In addition to the three mentioned regulations, there is also another regulation named the Waste Disposal (Chemical Waste) (General) Regulation (Cap.354C), which supervises the prohibition of improper packaging, labeling and storage of chemical wastes including asbestos before disposal since 1992 [19]. There are 4 regulations in total that regulate the asbestos operation in HK.

### 3.2. Organizations Concerning Asbestos Issue in HK and Their Actions on Asbestos Ban

HK government is not the only party which pays attention to asbestos issue but the “Positive Non-interventionism” policy implemented has kept the government in a relatively passive position in making policy changes. Comparatively, NGOs, labor unions and patients’ self-help organizations demonstrated a more aggressive and proactive attitude than the government and have played a key role in the development of asbestos banning policy in HK. Among all, Hong Kong Workers’ Health Centre (HKWHC) is one of the earliest and relatively proactive organizations to advocate for better protection and compensation for frontline industrial workers and also the general public.

HKWHC is a non-profit organization established by medical doctors, occupational health and safety professionals, rehabilitation therapists and social workers in 1984 [20]. Due to its multi-disciplinary background, compared to other interested parties, HKWHC has always been active in concerning different public health and occupational health issues including asbestos problems [21,22]. In addition to urge the government to develop a comprehensive compensation and rehabilitation support services for people in suffering from asbestosis and mesothelioma, HKWHC emphasized also the prevention of ARDs through primary prevention.

In 2009, a large-scale project in construction renovation sponsored by the HK government has started; buildings located in the urban area for over 30 years and lack maintenance are the target of the project [23]. Since the project also subsidized the cost of repair and maintenance work such as removal of rooftop structures and replacement of electrical wiring, the construction workers would face the hazard of asbestos when providing renovation work to old private buildings that contained asbestos substances. While the enforcement of regulations on renovation of private buildings relies on almost solely to the compliance of legal requirement of the property owners of these private buildings, protection and safeguarding to these construction workers may be inadequate.

To raise the awareness on the hazard and preventive measures of asbestos among the related construction workers, and also people who live nearby the suspected buildings under renovation and the waste collectors, HKWHC has collaborated with the largest local construction workers’ union, the Hong Kong Construction Industry Employees General Union (HKCIEGU), to start an educative project at community level since November 2011 [24]. This comprehensive health promotion intervention organized and provided numbers of health talks, exhibitions and carnivals accompanied with promotion leaflet delivered in the 5 districts condensed with the asbestos-containing buildings confirmed by the EPD, named the Kowloon City, Yau Tsim Mong, Sham Shui Po, Central and Western and Tsuen Wan District [25]. After organizing numbers of joint health talks and promotional activities with HKCIEGU and District Councilors to old building residents, the awareness of residents and also the councilors themselves were raised. Working together with HKCIEGU, more community-level activities were conducted since 2011, especially the messages about primary prevention to avoid improper handling of ACMs. Media coverage on the improper handling of ACMs and report of asbestos harm has raised further attention to this issue in HK. For instance, the sound-breaking ACM used inside rail vehicle in 2014 [22,26], the improper disposal of asbestos waste in rural area of Tsuen Wan in 2013 [27] and the asbestos cement sheet pieces scattered all around the construction site of Choi Yuen Village in 2011 [28,29], were the most spectacular news discovered and reported by NGOs, rather than the HK government.

One of the key achievements in this long journey for fighting for asbestos ban is that, even though there are ordinances to control the use of amosite and crocidolite and monitor their trading activity in Hong Kong since 1996, the lack of updated and further reaction from the government on total banning of asbestos was rather disappointing. When compared to other developed regimes which proactively establish a holistic system to a total ban of asbestos [13], as mentioned above, the action of Hong Kong government in this topic seems adapted with an evading attitude. Motivation was not seen in any total ban action and chrysotile or any substances made with it were still allowed to be imported into or re-exported in Hong Kong in 1996 to 2010. Active groups, including HKWHC, thus continued to lobby the idea of total banning of asbestos in HK and networked with legislative councilors to exert pressure on the government about the issue. After years of communication, lobbying, numerous petitions and meetings with the government representatives in the past decade, the EPD eventually changed its attitude and started to consider terminating the endless threat and burden from asbestos by amending the policy. On 20 April 2011, it submitted an administration paper to the Panel on Environmental Fair of the Legislative Council for the banning of all forms of asbestos [30]. In the proposal, the department proposed to extend the current banning policy on import and sale of blue and brown asbestos to all other forms of asbestos, and ban the supply and new use of all forms of asbestos through amending the Air Pollution Control Ordinance. This proposal has finally passed by the Legislative Council and enacted on 4 April 2014 [31]. The successful passage of this proposal is considered a great move in the passage of total banning of asbestos in the HK history, though the new clause of legislation only restricts any new use of ACMs and neglects the need for further planning on the elimination of existing ACMs in the community.

### 3.3. Development of Asbestos-Related Compensation System in HK

Other than the eventual change on regulation relating to the ban of asbestos mentioned above, the compensation system about ARDs has also undergone some changes by the effort of HKWHC, Pneumoconiosis Mutual Aid Association (PMAA) [32] and Association for the Rights of Industrial Accident Victims (ARIAV) [33].

At first, the British HK Government established a voluntary reporting system in 1956 for patients who suffered from asbestosis and other pneumoconiosis, and established the Pneumoconiosis Clinic to enforce the registration of cases and provision of medical assessment and curative services in 1974 [34].

Then on 20 April 1978, the government proposed to amend the labor law and established a compensation fund for workers diagnosed with pneumoconiosis. This proposal was passed by the Legislative Council and became the Pneumoconiosis Compensation Ordinance (Cap. 360) operated on 7 June 1978. However, due to the instability employment record of construction workers, the operation of the fund was not as good as expected. The government eventually stopped the fund and made a great modification after communicating with all concerned parties including construction industry unions, patients’ self-help organizations, non-governmental organizations (NGOs) and professionals. The reformed fund was launched again in 1980 [34]. Since then, the Pneumoconiosis Compensation Fund Board (PCFB) became the statutory body to be responsible for administering the fund that receives the resources from the levies of the construction and quarry industries in HK, and handles the compensation claim from any person suffering from pneumoconiosis and/or mesothelioma [35]. Apart from compensation, PCFB also has to finance programs for the prevention of pneumoconiosis and mesothelioma and those for the rehabilitation of patients suffering from those diseases.

Although there were more cases of ARDs reported after the compensation program reformed, the coverage of the fund was not comprehensive enough and resulting in large amount of surplus left in 1988 [36]. Due to the limitation under this policy, HKWHC linked with other patients’ networks, for instance PMAA, again and urged for a revision of Cap.360. The involved parties proposed that the amount of compensation on medical treatment expenses be increased, and the policy be expanded to cover also preventive and rehabilitation activities. No More Asbestos in Hong Kong Alliance (NMAHKA), an active body established by the ARIAV and Hong Kong Catholic Commission For Labor Affairs, also supported a reform of the policy and they particularly concerned for a comprehensive compensation process for patients and workers suffering from ARDs [37]. The actions taken by the active parties drew attention of the legislative councilors, labor unions and media. They then joined this campaign to pressure the government for a change.

Eventually, the government started to review the whole compensation program once again, and made several revisions in the following years, including changing to monthly compensation to patients rather than one-off payment since July 1993, re-classified the disability level in 1994 and adding “monthly compensation for pain, suffering and loss of amenities” into the compensable items in 1996 [34].

At 2007, HKWHC successfully lobbied the government to support the amendment of Pneumoconiosis Compensation Ordinance by incorporating the mesothelioma into the list of compensation [38]. The new ordinance was named as “Pneumoconiosis and Mesothelioma (Compensation) Ordinance”. The enactment of this amendment at 18 April 2008 made patients diagnosed with mesothelioma, as caused by inhalation of asbestos, eligible to receive the same compensations and benefits as patients diagnosed with silicosis and asbestosis [39].

## 4. Conclusions

It has not been an easy journey for all interested parties to fight for the total banning of asbestos in HK. Based on the experience we learnt from this asbestos battle through the years, efforts from different stakeholders including professional organizations, patients’ self-help organizations, NGOs, labor unions, and politicians are absolutely essential to the success of progression and development in today’s asbestos banning. Media attention and social power, on the other hand, is helpful and necessary in facilitating advocacy and prompting policy changes.

Banning the import and new use of asbestos is the first step to prevent asbestos exposure in Hong Kong. However, the amended regulation does not apply to existing ACMs in old construction structures. There is still room for amendment and improvement in current regulations and system for monitoring and managing existing asbestos. The mentioned parties will continue to strive for better revisions to root out existing asbestos, so as to make Hong Kong a true asbestos-free place for the elimination of ARDs.
